# Associations and Discriminatory Performance of Anthropometric and Body-Shape Indices for Identifying Hypertension in Adults with Type 2 Diabetes in Malawi: A Cross-Sectional Study

**DOI:** 10.3390/diagnostics16193083

**Published:** 2026-09-23

**Authors:** Alexander Thomas Mboma, Saulos Kondwani Nyirenda, Kondwani Joseph Banda, Alexander Archippus Kalimbira, Rong-Hong Hsieh, Tuyen Van Duong

**Affiliations:** 1 School of Nutrition and Health Sciences, College of Nutrition, Taipei Medical University, Taipei 11031, Taiwan; da07111005@tmu.edu.tw; 2 Department of Research and Development, Sub-Sahara Health and Research Institute, Lilongwe P.O. Box 40371, Malawi; s07691@tmu.edu.tw; 3 Internal Medicine Unit, Department of Medicine, Zomba Central Hospital, Zomba P.O. Box 21, Malawi; saulosnyirenda@yahoo.com; 4 School of Nursing, College of Nursing, Taipei Medical University, Taipei 11031, Taiwan; 5 Department of Human Nutrition and Health Sciences, Bunda College, Lilongwe University of Agriculture & Natural Resources, Lilongwe P.O. Box 219, Malawi; akalimbira@luanar.ac.mw

**Keywords:** type 2 diabetes, hypertension, anthropometry, central adiposity, waist-to-height ratio, waist circumference, body roundness index, body-shape indices, Malawi

## Abstract

**Background:** We evaluated the discriminatory performance of traditional anthropometric parameters and novel body-shape indices for identifying hypertension among adults with type 2 diabetes mellitus (T2DM) in Malawi. **Methods:** This was a multicenter cross-sectional study of 1356 adults with T2DM attending outpatient diabetes clinics at three tertiary referral hospitals. Traditional anthropometric parameters were body mass index (BMI), waist circumference (WC), and waist-to-height ratio (WHtR). Novel body-shape indices were the weight-adjusted waist index (WWI), a body shape index (ABSI), the conicity index, and body roundness index (BRI). Hypertension was defined as systolic blood pressure ≥ 140 mmHg and/or diastolic blood pressure ≥ 90 mmHg, or current antihypertensive medication use. Multivariable logistic regressions and receiver operating characteristic (ROC) analyses were performed overall and, as secondary subgroup analyses, stratified by sex; effect modification by sex was tested formally using interaction terms. **Results:** Overall, 68.3% had hypertension. All anthropometric measures were positively associated with hypertension in adjusted models. The strongest associations were observed for WHtR and its monotonic transformation, the BRI (aOR, 1.79; 95% CI 1.55–2.07 and aOR, 1.80; 95% CI 1.55–2.09, respectively), followed by WC (aOR, 1.70; 95% CI 1.48–1.95). The ranking of measures was similar in men and women, and no measure showed a statistically significant sex interaction. WHtR and BRI had the highest overall AUCs (0.69; 95% CI 0.66–0.72), followed by WC (0.68; 95% CI 0.65–0.71), indicating modest discrimination. Youden-derived WHtR cut-offs were 0.53 in men and 0.61 in women. **Conclusions:** All anthropometric measures were independently associated with hypertension, but discrimination was modest and none are adequate as a stand-alone screening test. WHtR is the simplest of the best-performing measures and may help prioritize patients for repeat blood pressure assessment in resource-limited diabetes clinics. The sex-stratified estimates and the internally derived cut-offs are exploratory and require external validation.

## 1. Introduction

Hypertension is a leading modifiable risk factor for cardiovascular morbidity and mortality worldwide, with a disproportionate burden among individuals with type 2 diabetes mellitus (T2DM) [1,2,3]. The coexistence of hypertension and T2DM accelerates the progression of cardiovascular disease, renal complications, and premature mortality [4,5], creating a major challenge for health systems, particularly in low- and middle-income countries (LMICs), where health system constraints amplify cardiometabolic risk [6,7]. In sub-Saharan Africa, rapid urbanization, dietary transitions, and increasing obesity have contributed to a rising burden of hypertension among people living with diabetes [8]. These trends highlight the need for effective, scalable, and context-appropriate approaches to cardiometabolic risk assessment.

Anthropometric measures are widely used in nutritional epidemiology and clinical practice to assess adiposity-related cardiometabolic risk. Traditional anthropometric parameters such as body mass index (BMI), waist circumference (WC), and waist-to-height ratio (WHtR) are simple and inexpensive but have important limitations [9]. BMI does not capture body fat distribution [9], and waist-based measures may not fully distinguish visceral from peripheral adiposity or account for variation in body shape [9]. These limitations are particularly relevant in individuals with T2DM, among whom central adiposity plays a critical role in the pathophysiology of hypertension and cardiovascular disease [10].

To address these limitations, several novel body-shape indices have been proposed to better characterize central fat accumulation and body geometry independently of overall body size. These include the weight-adjusted waist index (WWI) [11], a body shape index (ABSI) [12], the conicity index [13], and the body roundness index (BRI) [14]. Evidence suggests that these indices may improve the identification of cardiometabolic risk beyond BMI, including hypertension [15,16,17,18]. However, findings remain inconsistent across populations, and comparative evaluations of traditional anthropometric parameters and novel body-shape indices are limited, particularly in African settings where body composition patterns, nutritional exposures, and cardiometabolic risk profiles differ from those observed in high-income countries.

Sex-related differences in body fat distribution further complicate the interpretation of anthropometric indicators. Women generally exhibit higher total adiposity, whereas men are more prone to visceral fat accumulation [19], resulting in sex-specific patterns of hypertension risk [20]. Despite this, many studies rely on pooled analyses or uniform cut-off points across sexes, which may obscure meaningful differences in risk identification. Sex-stratified analyses using empirically derived thresholds may therefore help establish whether anthropometric risk assessment performs comparably in men and women.

Anthropometric assessment may complement routine blood pressure measurement by providing simple measures of adiposity-related cardiometabolic risk [9,15,16]. Because adiposity is a modifiable cardiovascular risk factor, identifying anthropometric indices that best discriminate hypertension may support risk stratification and lifestyle management in diabetes care.

This study examined the associations and comparative discriminatory performance of traditional anthropometric parameters and novel body-shape indices for identifying hypertension among adults with T2DM in Malawi, overall and by sex. Specifically, we evaluated traditional anthropometric parameters (BMI, WC, WHtR) and novel body-shape indices (WWI, ABSI, the conicity index, and BRI). We assessed their associations with hypertension using standardized continuous models and compared their discriminatory performance using ROC analysis with empirically derived optimal cut-off points, and pairwise AUC comparisons. We hypothesized that central adiposity measures, particularly WHtR, BRI, and WC, would demonstrate better discriminatory performance for hypertension than BMI. Sex-stratified analyses were conducted as secondary subgroup analyses, and any difference in optimal cut-off values between men and women was treated as exploratory.

## 2. Materials and Methods

### 2.1. Study Design and Setting

This multicenter cross-sectional study was conducted between November 2024 and January 2025 among adults with T2DM attending routine outpatient diabetes clinics at three tertiary referral hospitals in Malawi. The hospitals were purposively selected to represent all three administrative regions of Malawi: Mzuzu Central Hospital (Northern Region), Kamuzu Central Hospital (Central Region), and Zomba Central Hospital (Southern Region). These government-operated referral hospitals are located in urban areas and provide specialist outpatient care for chronic conditions, including diabetes. Data collection was embedded within routine clinic visits.

### 2.2. Study Population and Eligibility Criteria

Adults aged 18 years or older with a confirmed diagnosis of T2DM and at least six months of follow-up care were eligible. Participants were recruited consecutively during clinic attendance over the study period. We excluded individuals with type 1 diabetes, gestational diabetes, or prediabetes; pregnant women; individuals who were critically ill or hospitalized at the time of data collection; those with cognitive impairment precluding informed consent; and those with missing key hypertension outcome information (diagnosis status, antihypertensive medication records, or clinic-day blood pressure measurements). After applying these criteria, 1356 participants (494 men and 862 women) were included in the final analysis.

### 2.3. Sample Size Considerations

A formal a priori sample size calculation was not performed because recruitment was based on consecutive clinic attendance. However, the achieved sample size (1356 participants) provided sufficient numbers of events for sex-stratified analyses and multivariable modeling, given the high prevalence of hypertension in the study population. Because the limiting quantity for logistic models is the smaller outcome group, events-per-variable ratios were calculated using the number of non-hypertensive participants: 430 overall, 190 among men, and 240 among women. With one anthropometric measure and the covariates described in Section 2.5, all three strata retained a ratio above the conventional minimum of ten [21].

### 2.4. Data Collection Procedures and Quality Assurance

Data were collected by trained nurse research assistants using a pretested, interviewer-administered questionnaire and standardized anthropometric measurement protocols. All data collectors received uniform training prior to study initiation. Measurement equipment was calibrated across study sites, and standardized positioning and measurement procedures were applied consistently. Anthropometric measurements were taken once per participant; if a measurement appeared implausible, it was repeated and the second value recorded.

#### 2.4.1. Sociodemographic, Clinical, and Lifestyle Variables

Sociodemographic data included age (18–39, 40–59, 60–85 years), sex (male, female), marital status (never married/ever married), education level (pre-primary, primary, secondary, tertiary), and monthly household income, categorized as ≤150,000 vs. >150,000 Malawian Kwacha. The monthly income threshold approximated the median income distribution within the study sample and aligns with commonly used categorizations in Malawian socioeconomic research [22].

Clinical variables included duration of diabetes (<2 vs. ≥2 years), current use of antidiabetic medication, and glycemic control status. Glycemic control was classified using fasting blood glucose recorded on the day of data collection and categorized as optimal plasma glucose (70–130 mg/dL) or suboptimal plasma glucose (<70 mg/dL or >130 mg/dL). HbA1c was not routinely available across study sites and was therefore not used.

Lifestyle factors were assessed using structured questionnaires. Physical activity was measured using the International Physical Activity Questionnaire-Short Form (IPAQ-SF) [23]. The IPAQ-SF assesses walking, moderate-intensity activity, vigorous-intensity activity, and sitting time during the previous seven days. Physical activity was expressed as metabolic equivalent task minutes per week (MET-min/week) using the IPAQ scoring protocol [24]: walking MET-min/week = 3.3 × walking minutes × walking days; moderate-intensity MET-min/week = 4.0 × moderate-intensity minutes × moderate-intensity days; and vigorous-intensity MET-min/week = 8.0 × vigorous-intensity minutes × vigorous-intensity days. Total physical activity was calculated as the sum of walking, moderate-intensity, and vigorous-intensity MET-min/week. Total physical activity was then dichotomized as ≥600 versus <600 MET-min/week, the threshold corresponding to the World Health Organization (WHO) recommendation of at least 150 min of moderate-intensity activity, 75 min of vigorous-intensity activity, or an equivalent combination per week [24]. Physical activity was retained as a covariate because it is associated with both adiposity and blood pressure and is therefore a potential confounder of the association under study, rather than a variable of primary interest. Because it served only for adjustment, physical activity was entered in this binary form rather than as the three IPAQ categories (low, moderate, high) or as a continuous MET-min/week. The dichotomy was not intended to replace standard IPAQ categorical scoring or to preserve the full information contained in the continuous MET-min/week distribution. The ≥600 MET-min/week threshold approximates the boundary between the IPAQ low and moderate categories, and self-reported IPAQ-SF volumes have shown only modest criterion validity against objective measures [23], which limits the precision that continuous MET-min/week values would add. The ≥600 MET-min/week criterion is also the form in which physical activity is specified in the WHO guideline applied in the participating clinics. The implications of this coding are considered in Section 4.6.

Alcohol use and night eating were recorded as binary variables (yes/no). Night eating was assessed based on the presence of night eating behaviors in the previous seven days, defined as a composite of eating within two hours of bedtime and snacks after dinner [25]. Night eating was included as a covariate because evidence links circadian eating patterns with cardiometabolic regulation, including blood pressure variability [26].

#### 2.4.2. Anthropometric Measurements and Indices

Anthropometric measurements were obtained using validated, calibrated equipment following standardized operating procedures. Standing height was measured to the nearest 0.1 cm using a hospital-grade stadiometer, with participants barefoot and positioned according to WHO guidelines [27]. Body weight was measured to the nearest 0.1 kg using a calibrated digital scale, with participants wearing light clothing [27]. Waist circumference was measured at the midpoint between the lowest rib and the iliac crest using a non-stretchable measuring tape [28].

Traditional anthropometric parameters included BMI, WC, and WHtR. BMI was derived from weight and height, whereas WHtR was derived from waist circumference and height. Novel body-shape indices comprised WWI, ABSI, the conicity index, and BRI, calculated to capture central adiposity and body geometry independently of overall body size. Throughout the article, ‘anthropometric measures’ refers to all seven collectively.

*Traditional Anthropometric Parameters*

BMI was calculated as weight in kilograms divided by height in meters squared and categorized as <25 or ≥25 kg/m^2^ for descriptive purposes. Waist-to-height ratio (WHtR) was calculated as waist circumference in centimeters divided by height in centimeters. Although a WHtR ≥ 0.50 is commonly used as a general cardiometabolic risk threshold [29], this study also derived outcome-specific optimal cut-off points using ROC analysis.

*Novel Body-Shape Indices*

Novel body-shape indices were calculated using established formulas:

Weight-adjusted waist index (WWI) = WC (cm) ÷ √Weight (kg).

A body shape index (ABSI) = WC(m) ÷ (BMI^2/3^ × √Height (m)).

Conicity index = WC(m) ÷ [0.109 × √(Weight (kg)/Height (m))].

Body roundness index (BRI) = 364.2 − 365.5 × √[1 − (WC (m)/(2π))^2^ ÷ (0.5 × Heigh t(m))^2^].

Several of these measures are algebraically related, which constrains how their results can be interpreted. BRI is a strictly increasing function of WHtR, since WC (m)/(2π) ÷ (0.5 × Height (m)) reduces to WHtR/π; the two measures therefore rank participants identically and must produce the same area under the curve, sensitivity, specificity, and Youden’s index, with cut-offs that are exact transformations of one another. Similarly, the conicity index equals WWI × √Height (m) ÷ 0.109 and so differs from WWI only by a height-dependent scaling factor. Because height varies between participants, this relation is weaker than the WHtR–BRI identity: the two indices are highly, but not perfectly, concordant. These relations were taken into account when interpreting the correlation matrix, the pairwise AUC comparisons, and the incremental value of the more complex indices.

Because universally accepted clinical cut-offs are not available for several of these indices in African adults with T2DM, all seven anthropometric measures were analyzed both as standardized continuous variables and in relation to empirically derived thresholds.

#### 2.4.3. Outcome Definition

The primary outcome was hypertension, defined as systolic blood pressure ≥ 140 mmHg and/or diastolic blood pressure ≥ 90 mmHg recorded on the day of data collection, or current use of antihypertensive medication, following the threshold specified in the Seventh Report of the Joint National Committee (JNC 7) [30]. Blood pressure values used in this analysis were obtained from routine vital signs measurements conducted by clinic staff during the participants’ diabetes clinic visit on the day of data collection. The research team recorded the systolic and diastolic blood pressure values documented during routine clinic flow rather than conducting independent research-standardized blood pressure measurements. According to clinic protocols, blood pressure was measured with standard hospital automated sphygmomanometers after a brief rest while the participant was seated. The number of readings, cuff sizes, and exact rest periods were not systematically documented. Clinic-day blood pressure was therefore used to classify elevated blood pressure status for analytical purposes.

Participants with clinic blood pressure < 140/90 mmHg and no antihypertensive medication use were classified as non-hypertensive. As a sensitivity analysis, we used an expanded definition that additionally categorized participants with documented physician-diagnosed hypertension in the medical record as hypertensive. We retained the ≥140/90 mmHg threshold rather than the lower ≥130/80 mmHg threshold introduced by the 2017 ACC/AHA guideline [31] for three reasons. First, ≥140/90 mmHg remains the diagnostic threshold in the 2021 WHO guideline for the pharmacological treatment of hypertension [32], in the 2023 European Society of Hypertension guideline [33], and in Malawian national guidance for non-communicable disease care [34], so it is the threshold under which the participating clinics operate. Second, classification here rested on a single routine clinic reading, and a lower threshold applied to an unrepeated measurement would substantially increase false-positive classification. Third, ≥140/90 mmHg preserves comparability with the African diabetes literature against which our prevalence estimate is benchmarked. As an additional sensitivity analysis, all regression and ROC models were repeated using the ≥130/80 mmHg threshold (Supplementary Table S10).

### 2.5. Statistical Analysis

All anthropometric measures were analyzed using two complementary coding approaches. First, each measure was standardized into z-scores using the overall sample mean and standard deviation to estimate the odds of hypertension per 1-SD increase. The overall mean and standard deviation were also used in sex-stratified models, so that estimates for men and women are expressed on a common absolute scale and remain directly comparable. Second, sex-specific quartiles were derived for BMI, WC, WHtR, WWI, ABSI, the conicity index, and BRI using empirical percentile distributions, with the lowest quartile serving as the reference group. Quartile cut-off values are presented in Supplementary Table S1A.

Descriptive statistics were used to summarize participant characteristics. Categorical variables are presented as frequencies and percentages, and continuous variables are summarized using medians and interquartile ranges. We assessed differences between hypertensive and non-hypertensive participants within each sex using Pearson’s chi-square test for categorical variables and the Mann–Whitney U test for continuous variables.

Pearson correlation coefficients were calculated to assess linear relationships among anthropometric measures, to inform model specification and guard against multicollinearity (Supplementary Table S1B). Strong positive correlations were observed among waist-derived measures (e.g., WHtR with BRI, r = 0.99; WWI with conicity index, r = 0.98), while ABSI was inversely correlated with BMI (r = −0.29) consistent with its design to capture body shape independently of overall adiposity. Because of these strong pairwise correlations, each anthropometric measure was entered in a separate regression model to avoid multicollinearity, particularly between waist circumference and waist-to-height ratio.

Multivariable logistic regression models were fitted to estimate odds ratios (ORs) and 95% confidence intervals (CIs) for hypertension, overall and stratified by sex. Each anthropometric measure was entered in a separate regression model to avoid multicollinearity among closely related adiposity measures, particularly waist circumference and waist-to-height ratio. Covariates entered into the overall, men-specific and women-specific multivariable models were selected from an a priori candidate list drawn from the literature on determinants of hypertension in African populations with T2DM (age, sex, marital status, education, household income, duration of disease, glycemic control, alcohol use, night eating, and physical activity), retaining those supported by both ethe corresponding bivariate analysis and biological plausibility, with sex-specific factors entered only into the corresponding stratified models.

Overall models were adjusted for sex, age category, marital status, education, night eating, and physical activity. Men-specific models were adjusted for age category, marital status, monthly income, glycemic control, night eating, and physical activity. Women-specific models were adjusted for age category, marital status, education, and physical activity. BMI category was not included as a covariate because BMI is itself one of the exposures under evaluation, and adjusting for it would remove part of the adiposity signal that the remaining measures are intended to capture. Tests for linear trend across quartiles were conducted by modeling quartile medians as continuous variables. Quartile-based models are presented in Supplementary Table S2.

Because the bivariate associations differed between men and women, this procedure yielded different adjustment sets in the overall, male, and female models. To confirm that the ranking of anthropometric measures was not an artifact of differential adjustment, all models were repeated using a single common adjustment set applied to all three strata (age category, marital status, education, household income, glycemic control, night eating, and physical activity) (Supplementary Table S8).

Sex-stratified analyses were prespecified as secondary subgroup analyses, given established sex differences in body fat distribution [19]; formal interaction tests were used to determine whether any differences between strata exceeded those expected by chance. The interaction term addresses whether the strength of association differs by sex. Whether a single anthropometric threshold performs equivalently in men and women is a separate question, governed by the sex-specific distributions of the measure and of the outcome rather than by the odds ratio, and cut-offs were therefore derived within each sex regardless of the interaction result. The sample was not sized to detect modest effect modification, so the interaction results indicate the magnitude of modification the data can exclude rather than establishing its absence. Effect modification by sex was assessed by refitting each overall model with a product term between sex and the standardized anthropometric measure; the *p*-value for the product term is reported in Supplementary Table S3. Because physical activity may also modify the relation between adiposity and blood pressure, product terms between the physical activity indicator (≥600 versus <600 MET-min/week) and each standardized anthropometric measure were tested in the same way (Supplementary Table S9). The incremental contribution of each anthropometric measure was quantified by likelihood ratio tests comparing the covariate-only model with the model additionally containing the standardized measure, together with the corresponding change in AUC (Supplementary Table S4).

Discriminatory performance was evaluated using receiver operating characteristic (ROC) curve analysis. We calculated area under the curve (AUC) values with 95% confidence intervals for each anthropometric measure, overall and stratified by sex. Optimal cut-off points were identified using the maximum Youden’s index (sensitivity + specificity − 1) and interpreted as study-derived screening thresholds for identifying hypertension rather than diagnostic thresholds or replacements for conventional obesity classifications. As a complementary approach, cut-off points were also derived using the closest-to-(0,1) ROC criterion, defined as the point with the minimum Euclidean distance from the ideal ROC coordinate, where sensitivity = 1 and 1 − specificity = 0. Cut-offs from both methods were compared overall and stratified by sex. Pairwise comparisons of AUC values between anthropometric measures were conducted using DeLong tests for correlated ROC curves, overall and stratified by sex. Multiplicity was controlled using the Benjamini–Hochberg false discovery rate procedure at q = 0.05. The pairwise comparisons among the seven measures were treated as a single family, and the procedure was applied separately within each of the three analysis groups (overall, men, and women), giving three families of 21 tests. The WHtR-versus-BRI comparison carries no information, because the two measures generate the same ROC curve by construction. Sensitivity analyses were conducted using the expanded outcome definition that included documented physician-diagnosed hypertension. Descriptive, regression, and ROC analyses were conducted using IBM SPSS Statistics version 27.0. Pairwise DeLong comparisons were conducted using the pROC package in R version 4.5.3 (R Foundation for Statistical Computing, Vienna, Austria) using RStudio Desktop version 2026.01.1+403 (Posit Software, PBC, Boston, MA, USA), with two-sided *p* < 0.05 considered statistically significant.

## 3. Results

### 3.1. Participant Characteristics

A total of 1356 adults with T2DM were included, comprising 494 men (36.4%) and 862 women (63.6%). Using the standard definition based on measured clinic blood pressure or current antihypertensive medication use, 926 participants (68.3%) were classified as hypertensive and 430 (31.7%) as non-hypertensive. Overall, 819 participants (60.4%) had elevated clinic blood pressure on the day of data collection and 457 (33.7%) were using antihypertensive medication. Based on treatment and clinic blood pressure status, 430 participants (31.7%) had no antihypertensive medication use and clinic blood pressure < 140/90 mmHg, 107 (7.9%) were using antihypertensive medication with clinic blood pressure < 140/90 mmHg, 469 (34.6%) had no antihypertensive medication use but clinic blood pressure ≥ 140/90 mmHg, and 350 (25.8%) were using antihypertensive medication with clinic blood pressure ≥ 140/90 mmHg.

In the overall sample and in both sex-stratified analyses, hypertension was significantly associated with older age, being ever married, and physical activity below 600 MET-min/week. Education level, monthly income, duration of diabetes, glycemic control, alcohol use, and night eating were not significantly associated with hypertension in the overall analysis.

Across the overall sample and within both sexes, participants with hypertension had significantly higher median BMI, waist circumference, WHtR, WWI, ABSI, conicity index, and BRI than those without hypertension (Table 1).

### 3.2. Associations Between Traditional Anthropometric Parameters, Novel Body-Shape Indices, and Hypertension, Overall and by Sex

Correlation coefficients among all anthropometric measures are presented in Supplementary Table S1B; strong pairwise correlations supported modeling each measure separately (see Methods). In crude and fully adjusted logistic regression models, all seven anthropometric measures were positively associated with hypertension in the overall sample. In the overall adjusted models, WHtR and BRI showed the strongest associations, each 1-SD increase being associated with approximately 80% higher odds of hypertension (WHtR aOR 1.79, 95% CI: 1.55–2.07; BRI aOR 1.80, 95% CI: 1.55–2.09); the difference of 0.01 between them reflects the non-linear rescaling of WHtR into BRI rather than additional information. Strong associations were also observed for WC (aOR 1.70, 95% CI: 1.48–1.95), and BMI (aOR 1.50, 95% CI: 1.31–1.72). WWI, conicity index, and ABSI were also independently associated with hypertension, with adjusted ORs of 1.46 (95% CI: 1.28–1.67), 1.40 (95% CI: 1.23–1.60), and 1.24 (95% CI: 1.09–1.41), respectively (Table 2). Each anthropometric measure also produced a statistically significant improvement in model fit when added individually to the base covariate model, with the largest incremental gains observed for WHtR and BRI (likelihood ratio χ^2^(1) = 69.3 and 66.1, respectively; model AUC rising from 0.667 with covariates alone to 0.720; Supplementary Table S4).

In sex-stratified analyses, BRI and WHtR showed the strongest adjusted associations. Among men, adjusted ORs were 1.87 (95% CI: 1.43–2.43) for BRI, 1.84 (95% CI: 1.44–2.35) for WHtR, 1.67 (95% CI: 1.30–2.14) for BMI, 1.67 (95% CI: 1.33–2.11) for WC, 1.43 (95% CI: 1.15–1.78) for WWI, 1.34 (95% CI: 1.09–1.66) for conicity index, and 1.19 (95% CI: 0.97–1.46) for ABSI. Among women, corresponding adjusted ORs were 1.78 (95% CI: 1.48–2.13) for WHtR, 1.77 (95% CI: 1.47–2.13) for BRI, 1.73 (95% CI: 1.45–2.05) for WC, 1.48 (95% CI: 1.25–1.75) for WWI, 1.44 (95% CI: 1.21–1.70) for conicity index, 1.42 (95% CI: 1.20–1.69) for BMI, and 1.27 (95% CI: 1.08–1.49) for ABSI (Table 2).

Quartile-based analyses supported a graded increase in the odds of hypertension across sex-specific quartiles of anthropometric measures. For several measures, especially WHtR, BRI, WC, WWI, and conicity index, participants in higher quartiles generally had higher odds of hypertension than those in the lowest quartile (Supplementary Table S2). These results support a dose–response pattern, with progressively higher odds of hypertension at more adverse adiposity or body-shape profiles, although the cross-sectional design precludes interpretation as temporal risk progression.

No statistically significant sex interaction was detected for any of the seven anthropometric measures (all *p* for interaction ≥ 0.26; Supplementary Table S3). The sex-stratified estimates reported above are therefore descriptive subgroup findings and do not constitute evidence of effect modification by sex.

Repeating every model with a single adjustment set applied to all three strata (age category, marital status, education, household income, glycemic control, night eating, and physical activity) reproduced the primary estimates almost exactly: no adjusted odds ratio changed by more than 0.01 and the ranking of measures was unchanged in the overall sample and in both sexes (Supplementary Table S8). Product terms between the physical activity indicator and each standardized anthropometric measure were not statistically significant for any measure (all *p* ≥ 0.065; Supplementary Table S9). Adjusted associations were numerically weaker among participants with <600 MET-min/week than among those with ≥600 MET-min/week (for WHtR, aOR 1.49, 95% CI 1.00–2.22 versus aOR 1.87, 95% CI 1.60–2.18), but only 190 participants fell into the former group and the interaction tests do not support a genuine difference.

### 3.3. Discriminatory Performance of Traditional Anthropometric Parameters and Novel Body-Shape Indices

In ROC analysis, all anthropometric measures showed modest discriminatory performance for identifying hypertension, with AUC values ranging up to 0.69 (Table 3). In the overall sample, WHtR and BRI showed the highest AUC values, both 0.69 (95% CI: 0.66–0.72), followed by WC with an AUC of 0.68 (95% CI: 0.65–0.71). WHtR and BRI also had the highest Youden’s index values in the overall sample, both 0.27, with optimal cut-offs of 0.601 and 5.46, respectively. Because BRI is a strictly monotonic function of WHtR, their AUC, sensitivity, specificity, and Youden’s index values are identical by construction and their cut-offs are exact transformations of one another; the two are therefore reported together throughout rather than treated as independent measures. WC had an optimal cut-off of 96.75 cm, sensitivity of 49.2%, specificity of 76.5%, and Youden’s index of 0.26 (Table 3; Figure 1).

Among men, WHtR and BRI showed the highest AUC values, both 0.69 (95% CI: 0.65–0.74), and the highest Youden’s index values, both 0.31. Their optimal cut-offs were 0.53 for WHtR and 3.92 for BRI, with sensitivity of 71.7% and specificity of 58.9%. WC had an AUC of 0.67 (95% CI: 0.63–0.72) and an optimal cut-off of 84.50 cm (Table 3; Figure 1).

Among women, WHtR and BRI again showed the highest AUC values, both 0.67 (95% CI: 0.63–0.71), with Youden’s index values of 0.26. Their optimal cut-offs were 0.61 for WHtR and 5.54 for BRI. WC showed a similar AUC of 0.67 (95% CI: 0.63–0.71), with an optimal cut-off of 96.50 cm (Table 3; Figure 1).

Pairwise DeLong comparisons showed that WHtR and BRI had significantly higher AUCs than BMI, WWI, ABSI, and conicity index in the overall sample, but their AUCs did not differ significantly from WC. Of the 21 pairwise comparisons in the overall sample, 16 remained statistically significant after Benjamini–Hochberg correction, primarily those comparing WHtR or BRI with BMI and the other body-shape indices; the corresponding counts were 14 of 21 among women and 11 of the 20 testable comparisons among men. Among men, WHtR and BRI outperformed WWI, ABSI, and conicity index, but not BMI or WC after false discovery rate correction. The WHtR versus BRI comparison is not informative, because the two measures generate the same ROC curve by construction. Pairwise AUC comparisons are presented in Supplementary Table S6.

In sensitivity analysis using the expanded definition that additionally included documented hypertension diagnosis in the medical record, discriminatory performance increased modestly; WHtR and BRI had AUC values of 0.73 (95% CI: 0.70–0.76), compared with 0.69 (95% CI 0.66–0.72) under the primary definition, supporting the robustness of the main pattern (Supplementary Table S5A,B). In corresponding sensitivity logistic models, the direction and hierarchy of the associations remained consistent, with WHtR and BRI retaining the strongest associations with the expanded hypertension definition (Supplementary Table S5A,B). Applying the 2017 ACC/AHA threshold of ≥130/80 mmHg classified 1120 participants (82.6%) as hypertensive; adjusted associations and AUC values were essentially unchanged (for WHtR, aOR 1.81, 95% CI 1.51–2.17, and AUC 0.69), and the difference between the male and female Youden-derived WHtR cut-offs persisted (0.53 versus 0.61) (Supplementary Table S10).

Cut-offs derived using the closest-to-(0,1) criterion were compared with those derived using the maximum Youden’s index. Figure 2 visually summarizes the selected cut-off points from both approaches, and full comparisons are presented in Supplementary Table S7.

## 4. Discussion

In this multicenter study of adults with T2DM in Malawi, we evaluated the associations and discriminatory performance of traditional anthropometric parameters and novel body-shape indices for identifying hypertension based on clinic blood pressure and antihypertensive medication use. Four key findings emerged. First, hypertension was highly prevalent, affecting more than two-thirds of the participants. Second, all seven anthropometric measures were independently associated with hypertension in the overall adjusted analysis. Third, BRI and WHtR showed the strongest adjusted associations, suggesting that waist-height and body-roundness measures capture hypertension-relevant adiposity more effectively than BMI alone, although BRI is a deterministic transformation of WHtR and adds no information beyond it. Fourth, WHtR, BRI, and WC demonstrated the strongest discriminatory performance overall, with WHtR and BRI outperforming BMI, WWI, ABSI, and conicity index in pairwise comparisons, while remaining comparable to WC.

### 4.1. Participant Characteristics and Hypertension Status

The observed prevalence of hypertension (68.3%) underscores the substantial cardiometabolic burden among adults with T2DM in Malawi. This finding is consistent with evidence from other African diabetic populations, where hypertension is highly prevalent among people living with diabetes at 58.1% overall, with regional estimates of 77.6% in Central Africa, 69.1% in Southern Africa, 63.4% in North Africa, 51.5% in West Africa, and 53.0% in East Africa [35], reflecting the compounding effects of aging, urbanization, dietary transition, and rising obesity rates. Consistent with prior literature [36,37,38], hypertensive participants in both sexes were older and exhibited significantly higher levels of general and central adiposity than their non-hypertensive counterparts. These findings reinforce the central role of excess and centrally distributed adiposity in hypertension among individuals with T2DM, a group already at elevated cardiovascular risk.

Within both hypertension strata, median values of all seven anthropometric measures were higher in women than in men (Table 1), although no formal between-sex comparison was undertaken. These differences are consistent with other studies [39] and may reflect sex-specific differences in body composition and fat distribution, with women accumulating greater overall and subcutaneous adiposity, while men tend to accrue relatively more visceral fat at lower absolute levels of adiposity. Such differences motivated the secondary sex-stratified analyses reported below.

### 4.2. Independent Associations of Traditional Anthropometric Parameters and Novel Body-Shape Indices with Hypertension

Among the seven anthropometric measures, several waist-derived body-shape measures were strongly intercorrelated, particularly WHtR with BRI, WWI with conicity index, WWI with ABSI, and conicity index with ABSI. ABSI was only weakly and inversely correlated with BMI, which is consistent with its design to capture body-shape independently of overall adiposity [40,41]. Because of this overlap, each indicator was modeled in a separate multivariable regression rather than jointly, preserving the interpretability of each per 1-SD association while avoiding the inflated standard errors that multicollinearity would introduce.

In fully adjusted continuous models, all anthropometric measures were significantly associated with higher odds of hypertension in the overall sample. BRI and WHtR showed the strongest associations overall and in sex-stratified models, followed by WC and BMI, while WWI, conicity index, and ABSI also remained positively associated with hypertension. Consistent with previous studies, BRI has been reported as a strong anthropometric marker of cardiometabolic risk [42,43,44,45]. These associations persisted after adjustment for sociodemographic, lifestyle, and clinical factors, suggesting that traditional waist-derived parameters and novel body-shape indices capture dimensions of adiposity relevant to hypertension beyond basic demographic risk profiles [46].

These findings are biologically plausible. Central adiposity and unfavorable body geometry are closely linked to visceral fat accumulation, insulin resistance, chronic low-grade inflammation, and arterial stiffness, pathways that are particularly pronounced in individuals with T2DM [47,48]. WHtR and BRI, both derived from waist circumference and height, may therefore reflect hypertension-related central adiposity more closely than BMI alone. The findings should be interpreted as associations rather than causal effects because of the cross-sectional design.

### 4.3. Dose–Response Relationships Across Anthropometric Quartiles

Quartile-based analyses further supported a graded association between anthropometric measures and hypertension (Supplementary Table S2). For several measures, hypertension odds increased across higher quartiles, supporting a dose–response pattern. These gradients are consistent with previous evidence linking adverse anthropometric profiles with hypertension [49,50].

The most consistent quartile-based patterns were observed for BRI and WHtR, which also showed the strongest adjusted continuous associations. This supports the potential value of waist-height and body-roundness measures for cardiometabolic risk stratification. However, these quartile-based findings should be interpreted cautiously because the thresholds were internally derived and the cross-sectional design does not establish temporal risk progression.

### 4.4. Discriminatory Performance and Cut-Offs

Despite significant associations, none of the anthropometric measures achieved excellent discriminatory performance (AUC > 0.80) for hypertension. Discriminatory performance was modest, reflecting the multifactorial etiology of hypertension, which is influenced by age, genetics, renal function, diet, physical activity, medication adherence, glycemic control, and other metabolic factors beyond adiposity [51,52]. These findings underline that anthropometric measures cannot replace blood pressure measurement for diagnosing hypertension; rather, they may serve as adjunctive screening tools to prioritize individuals for more intensive blood pressure assessment and lifestyle counseling, particularly where resources are constrained. The AUC values observed here (0.58 to 0.69) correspond to poor-to-modest discrimination, and no measure reached the conventional 0.70 threshold for acceptable discrimination in any stratum. This interpretation is further tempered by the outcome definition: hypertension status rested on a single routine clinic reading combined with current medication use, and any resulting misclassification would be expected to attenuate the observed AUCs and to widen the uncertainty around the position of the ROC-derived cut-offs. Nevertheless, consistent with other studies [53,54,55], WHtR, BRI, and WC showed the most favorable screening performance.

In the overall sample, WHtR and BRI had identical AUC and Youden’s index values, while WC also performed well. Among men and women, WHtR and BRI consistently showed the highest or joint-highest AUC values. The identical performance of WHtR and BRI is not an empirical finding but an algebraic necessity: BRI is a strictly increasing function of WHtR, so the two impose the same ordering on participants and must return the same AUC, sensitivity, specificity, and Youden’s index. A comparable, if slightly weaker, redundancy holds between the conicity index and WWI, which differ only by a height-dependent scaling factor. From a practical perspective, WHtR may be especially attractive in resource-limited clinical settings, as it can be calculated and interpreted using simple waist-to-height comparisons without electronic tools, while providing a discriminatory performance comparable to BRI. Because BRI requires a non-linear formula and a calculator while offering no discriminatory gain over a ratio that clinic staff can compute mentally, we regard WHtR as the measure of choice and report BRI only for comparability with the existing literature. More broadly, the seven measures examined here represent fewer than seven distinct constructs, and the proliferation of novel indices should not be mistaken for additional information. The pairwise DeLong results reinforce this interpretation. WHtR and BRI discriminated hypertension better than BMI and the other body-shape indices with weaker AUCs, but they did not significantly outperform WC. This suggests that waist-based measures are useful for screening, but the practical advantage of more complex derived indices over simple waist circumference may be limited in routine clinical settings.

Two further checks support these conclusions. Applying a single common adjustment set across the overall, male, and female models left every adjusted odds ratio unchanged to within 0.01 (Supplementary Table S8), so the ranking of measures is not an artifact of the differing covariate sets. Testing for effect modification by physical activity, as distinct from sex, produced no significant interaction for any measure (Supplementary Table S9); adjusted associations were numerically weaker among the 190 participants with <600 MET-min/week, but that subgroup is too small to separate genuine attenuation from chance.

ABSI showed statistically significant associations with hypertension but relatively weaker discriminatory performance. This highlights the distinction between association and discrimination: an anthropometric measure may be independently associated with hypertension but still have limited value as a standalone screening tool.

The optimal cut-off points identified in this study differed from some conventional anthropometric thresholds. This difference is expected because conventional cut-offs are commonly used to classify general or central obesity in broader populations, whereas the present cut-offs were derived specifically to discriminate hypertension among adults with T2DM. Adults with T2DM already have a high baseline cardiometabolic risk, and the relationship between adiposity and hypertension may differ from that observed in the general population.

The BMI cut-off of 27.16 kg/m^2^ in the overall sample was higher than the conventional overweight threshold, so that applying the conventional ≥25 kg/m^2^ threshold in this population would classify a large proportion of participants as at risk while adding little discrimination. BMI also yielded lower AUCs than WHtR, BRI, and WC in every stratum. This is consistent with our earlier analysis in the same cohort, in which BMI-defined overweight/obesity and WHtR-defined central obesity agreed only partially, so that some participants with a BMI below 25 kg/m^2^ nonetheless met the WHtR criterion for central obesity [56].

These findings should not be interpreted as replacing established obesity or central obesity classifications. Instead, the reported thresholds should be viewed as study-derived screening cut-offs optimized for identifying hypertension in this specific T2DM population. Conventional WHtR thresholds such as 0.50 [57] remain useful for general cardiometabolic risk screening, whereas the higher WHtR cut-offs around 0.60 observed here may better reflect the risk gradient for prevalent hypertension among adults with T2DM. These cut-offs remain exploratory. They were derived internally in a cross-sectional sample drawn from three urban tertiary diabetes clinics, they shifted when the closest-to-(0,1) criterion was substituted for the Youden’s index (Supplementary Table S7), and Youden-derived thresholds are known to be unstable at this sample size. They should not be treated as validated clinical thresholds until they have been reproduced in independent African T2DM populations.

### 4.5. Sex-Stratified Findings

Sex-stratified analyses showed broadly consistent patterns, with BRI and WHtR showing the strongest adjusted associations in both sexes. Some numerical differences were observed: ABSI was significantly associated with hypertension among women but not among men after adjustment, and WHtR and BRI showed marginally higher AUCs among men than women, a pattern elsewhere attributed to hormonal, metabolic, and inflammatory differences [58,59,60].

These differences should not be over-interpreted. The formal sex-interaction tests were not statistically significant for any measure (Supplementary Table S3); the confidence intervals for the male and female AUCs overlap substantially (for WHtR, 0.65 to 0.74 in men versus 0.63 to 0.71 in women); and the non-significant ABSI estimate in men (aOR 1.19, 95% CI 0.97–1.46) is not statistically distinguishable from the significant estimate in women (aOR 1.27, 95% CI 1.08–1.49). The sex-stratified results are therefore presented as descriptive subgroup findings, with the smaller male stratum (*n* = 494) yielding less precise estimates than the female stratum (*n* = 862). Sex-specific cut-offs may nonetheless differ from a common cut-off for reasons unrelated to effect modification. An interaction term concerns the odds ratio per 1-SD increase; a cut-off concerns the point on each sex’s distribution at which sensitivity and specificity are balanced. Among non-hypertensive participants, median WHtR was 0.51 in men and 0.57 in women, and the median waist circumference 83.0 cm and 89.5 cm, respectively (Table 1). A threshold maximizing the Youden’s index in the pooled sample therefore falls at a different percentile in each sex and yields different sensitivity and specificity in each, even where the underlying association is identical. Power is a further consideration: with 190 non-hypertensive men and 240 non-hypertensive women, only substantial departures from a common odds ratio can be excluded, so the non-significant interaction terms constitute weak evidence of homogeneity rather than a demonstration of it.

Because the interaction tests were null, any mechanistic account of the numerical sex differences remains speculative. One mechanism proposed for the ABSI pattern is the estrogen-related differences in body fat patterning [61], and a second is that ABSI was originally derived in a US National Health and Nutrition Examination Survey population [62], which differs from sub-Saharan African populations in adiposity distribution. Notably, WHtR and BRI optimal cut-offs for hypertension were lower among men (0.53 and 3.92, respectively) than among women (0.61 and 5.54), opposite to the pattern reported in most prior literature from non-African populations [63]. This inversion may reflect African-specific differences in fat distribution [64], lean mass, and the relative threshold at which central adiposity confers cardiometabolic risk in men versus women with T2DM [65]. It is the observation most clearly at odds with the non-African literature, but the male and female cut-offs were estimated separately within each sex and the separation between them has not itself been formally tested against sampling variation. It is therefore offered as a hypothesis for testing in larger and preferably prospective African cohorts, not as a basis for adopting different thresholds for men and women in practice. The pattern was, however, reproduced when the ACC/AHA threshold was applied (Supplementary Table S10), so it is not an artifact of the blood pressure cut-point chosen. Together, these findings suggest that WHtR, BRI, and WC may serve as practical adjuncts to routine cardiometabolic risk assessment in adults with T2DM, pending external validation.

### 4.6. Strengths and Limitations

Key strengths of this study include its large multicenter clinical sample, standardized anthropometric assessment, comprehensive covariate adjustment, and pre-specified secondary sex-stratified analyses accompanied by formal tests of effect modification. The study also provides locally relevant ROC-derived cut-off points for multiple anthropometric measures in an underrepresented African T2DM population.

Several limitations should be considered. First, the cross-sectional design precludes causal inference and does not allow assessment of incident hypertension. Second, the sample included a larger proportion of women than men, which may reflect sex differences in clinic attendance or health-seeking behavior in outpatient diabetes care. Although sex-stratified analyses were conducted, the larger female sample may have influenced the overall estimates and produced more precise estimates among women than men. Therefore, the overall findings should be interpreted in light of the sex distribution of this study population. Third, blood pressure values were taken from routine clinic vital signs records rather than independent research-standardized measurements. Repeated BP measurements, rest-period standardization, cuff-size documentation, and averaging of multiple readings were not available for all participants. Some measurement variability and misclassification are therefore possible. However, the use of routinely recorded clinic BP reflects real-world diabetes clinic practice and is relevant for pragmatic cardiometabolic risk assessment in resource-limited settings. Fourth, antihypertensive medication use was derived from documented medical records in the health passports, so documentation errors cannot be excluded. Fifth, residual collinearity among closely related adiposity measures cannot be fully excluded, although each anthropometric measure was modeled separately. Sixth, although pairwise DeLong tests were performed, these comparisons should be interpreted cautiously because several anthropometric measures are mathematically related, particularly WHtR and BRI. Seventh, the ROC-derived cut-offs are internally derived and require external validation before clinical application.

Finally, several measurement and generalizability constraints apply. HbA1c was unavailable, so glycemic control was based on a single fasting glucose value, which is an imprecise proxy. Antihypertensive treatment lowers measured blood pressure, so treated participants with controlled pressure were classified as hypertensive on the basis of medication use alone; this is appropriate for prevalence but weakens the link between adiposity and the measured blood pressure that the ROC analysis exploits. Dietary sodium intake, medication adherence, and family history of hypertension were not captured and may confound the observed associations. Physical activity was entered as a binary indicator (≥600 versus <600 MET-min/week) rather than as IPAQ categories or continuous MET-min/week. Dichotomization discards information on the distribution of activity volume, may reduce the precision of confounding control, and may obscure graded associations between physical activity and hypertension. Any residual confounding by activity volume would be expected to bias the adjusted odds ratios away from the null, because higher activity is associated with both lower adiposity and lower blood pressure. This coding does not affect the single-measure ROC analyses, AUC values, or cut-offs (Table 3), which were estimated without covariate adjustment, and because the same coding was used in every model it is unlikely to have altered the ranking of measures. Anthropometric measurements were taken once per participant and inter-observer technical error of measurement was not quantified. In addition, recruitment was confined to urban tertiary referral clinics, so the findings may not extend to rural primary care settings, where most Malawian adults with T2DM receive care.

## 5. Conclusions

In this multicenter cross-sectional study of Malawian adults with T2DM, all seven anthropometric measures were independently associated with hypertension, most strongly WHtR and its monotonic transformation BRI, followed by WC. Discrimination was modest for every measure (AUC 0.58 to 0.69), and none reached the threshold conventionally regarded as acceptable for stand-alone screening. Youden-derived cut-offs differed between men and women (WHtR 0.53 versus 0.61) and from conventional thresholds, but the sex-interaction tests were not significant and these values were derived internally. The null interaction tests do not support inference of sex-specific effects or of sex-specific clinical thresholds. External validation in independent African T2DM populations, and prospective evaluation against incident hypertension, are required before any of these thresholds is applied clinically.

## Figures and Tables

**Figure 1:**
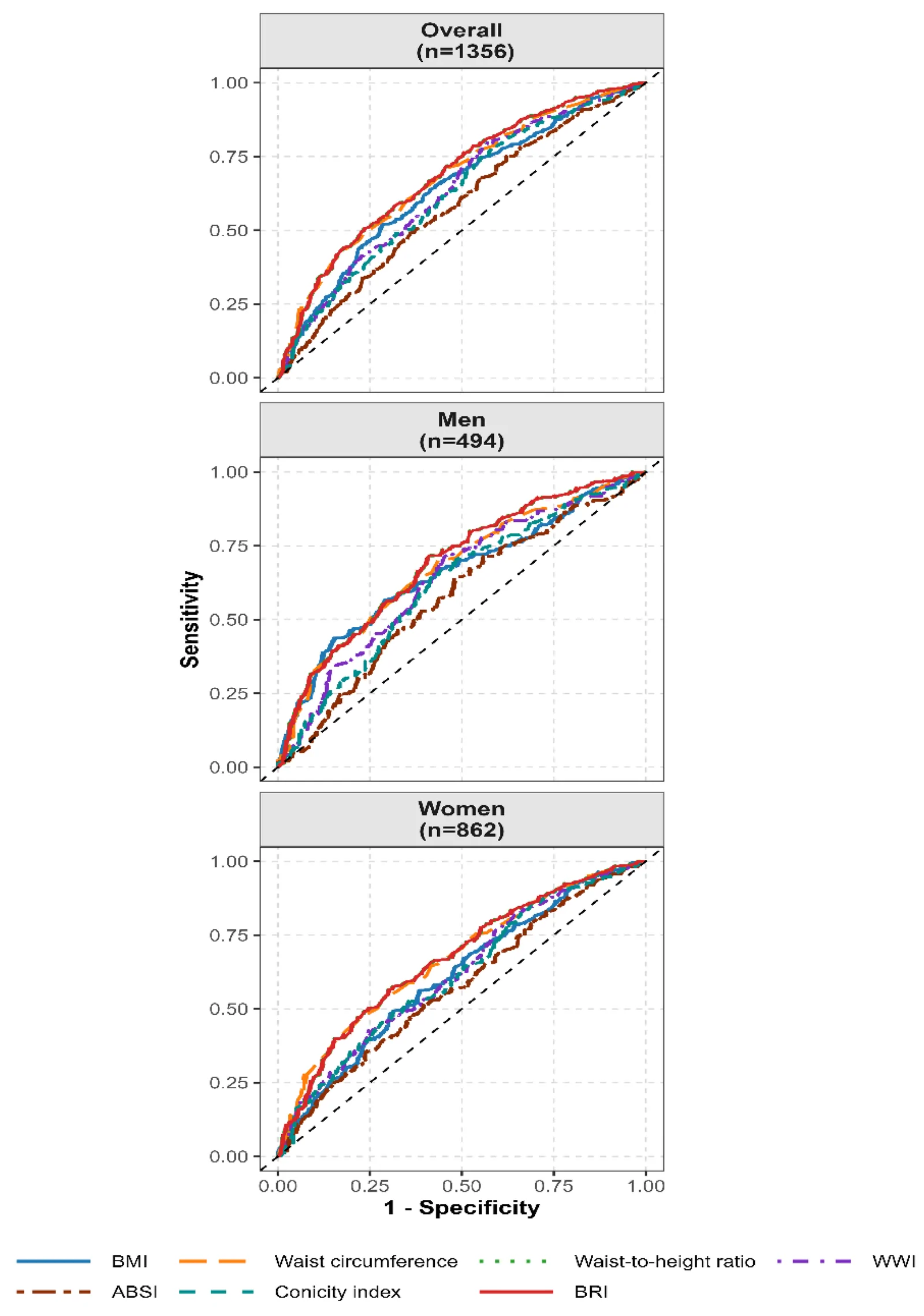
Receiver operating characteristic curves for traditional anthropometric parameters and novel body-shape indices identifying hypertension among adults with type 2 diabetes, overall and stratified by sex. Abbreviations: BMI, body mass index; WWI, weight-adjusted waist index; ABSI, a body-shape index; BRI, body roundness index.

**Figure 2:**
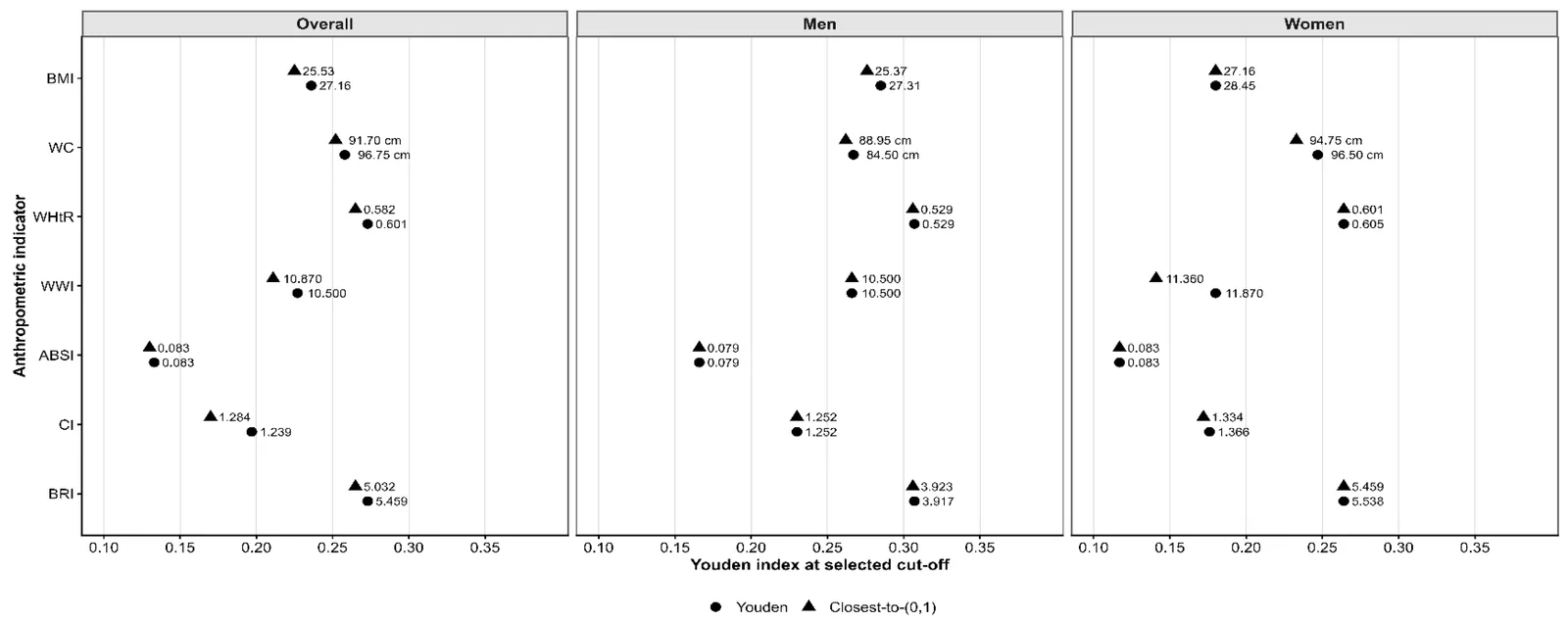
Comparison of optimal cut-off points derived using the maximum Youden’s index and the closest-to-(0,1) ROC criterion for traditional anthropometric parameters and novel body-shape indices identifying hypertension among adults with type 2 diabetes mellitus. Abbreviations: BMI, body mass index; WC, waist circumference; WHtR, waist-to-height ratio; WWI, weight-adjusted waist index; ABSI, a body shape index; BRI, body roundness index.

**Table 1:** Participant sociodemographic, clinical, lifestyle, and anthropometric characteristics by hypertension status among adults with type 2 diabetes mellitus (*n* = 1356).

Variable	Total (*N* = 1356)	Overall			Men			Women		
		No HTN (*n* = 430)	HTN (*n* = 926)	*p*	No HTN (*n* = 190)	HTN (*n* = 304)	*p*	No HTN (*n* = 240)	HTN (*n* = 622)	*p*
Age *n* (%)				<0.001			<0.001			<0.001
18–39 years	270 (19.9)	148 (34.4)	122 (13.2)		77 (40.5)	55 (18.1)		71 (29.6)	67 (10.8)	
40–59 years	575 (42.4)	171 (39.8)	404 (43.6)		68 (35.8)	118 (38.8)		103 (42.9)	286 (46.0)	
60–85 years	511 (37.7)	111 (25.8)	400 (43.2)		45 (23.7)	131 (43.1)		66 (27.5)	269 (43.2)	
Marital status, *n* (%)				<0.001			0.006			0.008
Never married	100 (7.4)	49 (11.4)	51 (5.5)		23 (12.1)	16 (5.3)		26 (10.8)	35 (5.6)	
Ever married	1256 (92.6)	381 (88.6)	875 (94.5)		167 (87.9)	288 (94.7)		214 (89.2)	587 (94.4)	
Education level, *n* (%)				0.158			0.573			0.105
Pre-primary	123 (9.1)	34 (7.9)	89 (9.6)		11 (5.8)	15 (4.9)		23 (9.6)	74 (11.9)	
Primary	561 (41.4)	168 (39.1)	393 (42.4)		68 (35.8)	97 (31.9)		100 (41.7)	296 (47.6)	
Secondary	528 (38.9)	186 (43.3)	342 (36.9)		88 (46.3)	143 (47.0)		98 (40.8)	199 (32.0)	
Tertiary	144 (10.6)	42 (9.8)	102 (11.0)		23 (12.1)	49 (16.1)		19 (7.9)	53 (8.5)	
Monthly income, MWK				0.175			0.108			0.293
≤150,000	931 (68.7)	306 (71.2)	625 (67.5)		125 (65.8)	178 (58.6)		181 (75.4)	447 (71.9)	
>150,000	425 (31.3)	124 (28.8)	301 (32.5)		65 (34.2)	126 (41.4)		59 (24.6)	175 (28.1)	
Duration of diabetes				0.158			0.593			0.248
<2 years	236 (17.4)	84 (19.5)	152 (16.4)		40 (21.1)	58 (19.1)		44 (18.3)	94 (15.1)	
≥2 years	1120 (82.6)	346 (80.5)	774 (83.6)		150 (78.9)	246 (80.9)		196 (81.7)	528 (84.9)	
Glycemic control				0.505			0.119			0.836
Suboptimal PG	907 (66.9)	293 (68.1)	614 (66.3)		130 (68.4)	187 (61.5)		163 (67.9)	427 (68.6)	
Optimal PG	449 (33.1)	137 (31.9)	312 (33.7)		60 (31.6)	117 (38.5)		77 (32.1)	195 (31.4)	
Alcohol use				0.825			0.725			0.857
No	1317 (97.1)	417 (97.0)	900 (97.2)		179 (94.2)	284 (93.4)		238 (99.2)	616 (99.0)	
Yes	39 (2.9)	13 (3.0)	26 (2.8)		11 (5.8)	20 (6.6)		2 (0.8)	6 (1.0)	
Night eating				0.062			0.279			0.116
No night eating	656 (48.4)	224 (52.1)	432 (46.7)		97 (51.1)	140 (46.1)		127 (52.9)	292 (46.9)	
Night eating	700 (51.6)	206 (47.9)	494 (53.3)		93 (48.9)	164 (53.9)		113 (47.1)	330 (53.1)	
Physical activity, MET-min/week				<0.001			0.017			0.020
≥600	1166 (86.0)	390 (90.7)	776 (83.8)		175 (92.1)	258 (84.9)		215 (89.6)	518 (83.3)	
<600	190 (14.0)	40 (9.3)	150 (16.2)		15 (7.9)	46 (15.1)		25 (10.4)	104 (16.7)	
Anthropometrics, median (IQR)										
**Traditional Anthropometric Parameters**										
BMI, kg/m^2^	26.40 (23.03–30.34)	24.78 (22.05–27.91)	27.47 (24.03–31.16)	<0.001	23.42 (21.62–26.01)	25.81 (22.62–29.88)	<0.001	26.02 (22.72–29.41)	28.01 (24.59–31.61)	<0.001
WC, cm	93.00 (83.00–103.00)	87.00 (78.00–96.00)	96.00 (86.00–106.00)	<0.001	83.00 (76.75–94.00)	93.00 (82.00–101.45)	<0.001	89.50 (80.00–98.00)	98.00 (88.00–108.00)	<0.001
WHtR	0.58 (0.52–0.66)	0.54 (0.47–0.60)	0.60 (0.54–0.68)	<0.001	0.51 (0.46–0.57)	0.57 (0.51–0.64)	<0.001	0.57 (0.50–0.62)	0.62 (0.56–0.69)	<0.001
**Novel Body-Shape Indices**										
WWI	11.33 (10.36–12.24)	10.86 (9.87–11.72)	11.46 (10.65–12.40)	<0.001	10.38 (9.67–11.42)	11.20 (10.26–12.01)	<0.001	11.23 (10.19–11.87)	11.63 (10.88–12.52)	<0.001
ABSI	0.082 (0.076–0.089)	0.080 (0.074–0.087)	0.083 (0.077–0.090)	<0.001	0.079 (0.074–0.086)	0.082 (0.076–0.089)	0.004	0.082 (0.075–0.087)	0.084 (0.077–0.091)	<0.001
Conicity index	1.31 (1.20–1.41)	1.26 (1.16–1.37)	1.33 (1.23–1.43)	<0.001	1.21 (1.15–1.35)	1.30 (1.19–1.40)	<0.001	1.29 (1.16–1.38)	1.34 (1.25–1.44)	<0.001
BRI	5.03 (3.64–6.76)	4.14 (2.90–5.43)	5.52 (4.15–7.24)	<0.001	3.55 (2.56–4.80)	4.77 (3.59–6.45)	<0.001	4.71 (3.37–5.91)	5.92 (4.48–7.54)	<0.001

Data are presented as *n* (%) for categorical variables and median (interquartile range, IQR) for continuous variables. *p*-values were obtained using Pearson’s chi-square test for categorical variables and Mann–Whitney U test for continuous variables. **Abbreviations**: MWK, Malawian Kwacha; BMI, body mass index; WC, waist circumference; WHtR, waist-to-height ratio; WWI, weight-adjusted waist index; ABSI, a body shape index; BRI, body roundness index.

**Table 2:** Crude and adjusted associations of traditional anthropometric parameters and novel body-shape indices with hypertension among adults with T2DM, overall and stratified by sex.

					Hypertension							
Parameter, per 1-SD Increase	Overall (*n* = 1356)				Men (*n* = 494)				Women (*n* = 862)			
	OR (95% CI)	*p*	aOR (95% CI)	*p*	OR (95% CI)	*p*	aOR (95% CI)	*p*	OR (95% CI)	*p*	aOR (95% CI)	*p*
**Traditional Anthropometric Parameters**												
**BMI**	1.74 (1.52–1.99)	<0.001	1.50 (1.31–1.72)	<0.001	2.00 (1.57–2.54)	<0.001	1.67 (1.30–2.14)	<0.001	1.55 (1.31–1.82)	<0.001	1.42 (1.20–1.69)	<0.001
**WC**	1.96 (1.72–2.23)	<0.001	1.70 (1.48–1.95)	<0.001	1.97 (1.59–2.45)	<0.001	1.67 (1.33–2.11)	<0.001	1.88 (1.59–2.22)	<0.001	1.73 (1.45–2.05)	<0.001
**WHtR**	2.06 (1.80–2.36)	<0.001	1.79 (1.55–2.07)	<0.001	2.17 (1.72–2.73)	<0.001	1.84 (1.44–2.35)	<0.001	1.94 (1.63–2.31)	<0.001	1.78 (1.48–2.13)	<0.001
**Novel Body-Shape Indices**												
**WWI**	1.63 (1.44–1.85)	<0.001	1.46 (1.28–1.67)	<0.001	1.63 (1.32–2.01)	<0.001	1.43 (1.15–1.78)	0.001	1.56 (1.32–1.83)	<0.001	1.48 (1.25–1.75)	<0.001
**ABSI**	1.31 (1.16–1.48)	<0.001	1.24 (1.09–1.41)	<0.001	1.27 (1.05–1.55)	0.016	1.19 (0.97–1.46)	0.090	1.31 (1.12–1.53)	<0.001	1.27 (1.08–1.49)	0.004
**Conicity index**	1.54 (1.36–1.75)	<0.001	1.40 (1.23–1.60)	<0.001	1.50 (1.23–1.84)	<0.001	1.34 (1.09–1.66)	0.006	1.51 (1.28–1.77)	<0.001	1.44 (1.21–1.70)	<0.001
**BRI**	2.09 (1.81–2.42)	<0.001	1.80 (1.55–2.09)	<0.001	2.25 (1.75–2.89)	<0.001	1.87 (1.43–2.43)	<0.001	1.94 (1.62–2.33)	<0.001	1.77 (1.47–2.13)	<0.001

Crude odds ratios were estimated from univariable logistic regression models. Adjusted odds ratios were estimated from separate multivariable logistic regression models for each anthropometric measure. Overall models were adjusted for sex, age category, marital status, education, night eating, and physical activity. Men-specific models were adjusted for age category, marital status, monthly income, glycemic control, night eating, and physical activity. Women-specific models were adjusted for age category, marital status, education, and physical activity. Odds ratios represent the odds of hypertension per one-standard deviation increase in each measure. Abbreviations: OR, odds ratio; aOR, adjusted odds ratio; CI, confidence interval; BMI, body mass index; WC, waist circumference; WHtR, waist-to-height ratio; WWI, weight-adjusted waist index; ABSI, a body shape index; BRI, body roundness index.

**Table 3:** Discriminatory performance and optimal cut-off points of traditional anthropometric parameters and novel body-shape indices for identifying hypertension among adults with T2DM (*n* = 1356).

Parameter	Overall					Men					Women				
	Cut-Off	Sens (%)	Spec (%)	Youden	AUC (95% CI)	Cut-Off	Sens (%)	Spec (%)	Youden	AUC (95% CI)	Cut-Off	Sens (%)	Spec (%)	Youden	AUC (95% CI)
**Traditional Anthropometric Parameters**															
**BMI**	27.16	52.2	71.4	0.236	0.643 (0.611–0.674)	27.31	43.8	84.7	0.285	0.657 (0.609–0.705)	28.45	48.4	69.6	0.180	0.612 (0.570–0.654)
**WC**	96.75	49.2	76.5	0.258	0.678 (0.648–0.708)	84.50	70.4	56.3	0.267	0.674 (0.626–0.722)	96.50	53.1	71.7	0.247	0.667 (0.628–0.707)
**WHtR**	0.601	50.8	76.5	0.273	0.688 (0.658–0.717)	0.529	71.7	58.9	0.307	0.692 (0.645–0.739)	0.605	56.4	70.0	0.264	0.670 (0.630–0.709)
**Novel Body-Shape Indices**															
**WWI**	10.50	79.7	43.0	0.227	0.635 (0.603–0.667)	10.50	71.4	55.3	0.266	0.640 (0.589–0.690)	11.87	42.6	75.4	0.180	0.618 (0.576–0.659)
**ABSI**	0.083	49.1	64.2	0.133	0.581 (0.549–0.614)	0.079	64.5	52.1	0.166	0.576 (0.524–0.628)	0.083	51.3	60.4	0.117	0.576 (0.534–0.618)
**Conicity index**	1.239	74.6	45.1	0.197	0.623 (0.591–0.655)	1.252	65.1	57.9	0.230	0.617 (0.566–0.668)	1.366	44.7	72.9	0.176	0.614 (0.573–0.656)
**BRI**	5.459	50.8	76.5	0.273	0.688 (0.658–0.717)	3.917	71.7	58.9	0.307	0.692 (0.645–0.739)	5.538	56.4	70.0	0.264	0.670 (0.630–0.709)

**Abbreviations**: Sens, sensitivity; Spec, specificity; AUC, area under the curve; CI, confidence interval; BMI, body mass index; WC, waist circumference; WHtR, waist-to-height ratio; WWI, weight-adjusted waist index; ABSI, a body shape index; BRI, body roundness index. Optimal cut-off values were determined using the maximum Youden’s index.

## Data Availability

The data presented in this study are available on request from the corresponding author due to ethical and privacy reasons.

## Supplementary Materials

The following supporting information can be downloaded at: https://www.mdpi.com/article/10.3390/diagnostics16193083/s1, Supplementary Table S1A. Sex-specific quartile ranges for traditional anthropometric parameters and novel body-shape indices among adults with type 2 diabetes mellitus; Supplementary Table S1B. Pearson correlation coefficients among traditional anthropometric parameters and novel body-shape indices among adults with type 2 diabetes mellitus; Supplementary Table S2. Adjusted associations between sex-specific quartiles of traditional anthropometric parameters, novel body-shape indices and hypertension among adults with type 2 diabetes mellitus; Supplementary Table S3. Sex interaction tests for associations of traditional anthropometric parameters and novel body-shape indices with hypertension among adults with type 2 diabetes mellitus; Supplementary Table S4. Incremental predictive value of traditional anthropometric parameters and novel body-shape indices for identifying hypertension among adults with type 2 diabetes mellitus; Supplementary Table S5A. Sensitivity analysis using expanded clinical hypertension definition: adjusted logistic regression; Supplementary Table S5B. Sensitivity ROC analysis using expanded clinical hypertension definition; Supplementary Table S6. Pairwise DeLong comparisons of AUC values between key anthropometric measures; Supplementary Table S7. Comparison of optimal cut-off points derived using maximum Youden index and closest-to-(0,1) ROC criterion; Supplementary Table S8. Sensitivity analysis of associations between anthropometric measures and hypertension using a common covariate adjustment set; Supplementary Table S9. Interaction between physical activity status and anthropometric measures in their associations with hypertension; Supplementary Table S10. Sensitivity analysis of anthropometric associations and discriminatory performance using the 2017 ACC/AHA hypertension threshold.
