# Supplementary material for: Associations and Discriminatory Performance of Anthropometric and Body-Shape Indices for Identifying Hypertension in Adults with Type 2 Diabetes in Malawi: A Cross-Sectional Study
Source: Diagnostics (Basel). 2026 Sep 23;16(19):3083. doi: 10.3390/diagnostics16193083 (PMC13664518; doi:10.3390/diagnostics16193083)
Supplement: Supplementary file 1 [file diagnostics-16-03083-s001.zip › diagnostics-4456620-supplementary.pdf]

Supplementary Material

Manuscript: Associations and Discriminatory Performance of Traditional Anthropometric Parameters and Novel Body-Shape Indices for Identifying Hypertension in Adults with Type 2 Diabetes in Malawi

Supplementary Table S1A. Sex-specific quartile ranges for traditional anthropometric parameters and novel body-shape indices among adults with type 2 diabetes mellitus.

Index	Men Q1	Men Q2	Men Q3	Men Q4	Women Q1	Women Q2	Women Q3	Women Q4
**BMI**	13.38-22.22	22.22-24.84	24.86-28.61	28.61-50.89	13.82-24.09	24.09-27.38	27.39-31.13	31.13-48.68
**Waist circumference**	62.0-79.0	79.0-89.0	89.0-99.0	99.0-130.0	62.0-85.0	85.0-95.0	95.0-105.0	105.0-130.0
**WHtR**	0.374-0.479	0.480-0.549	0.550-0.613	0.613-0.892	0.388-0.538	0.538-0.602	0.603-0.673	0.673-0.923
**WWI**	7.76-9.93	9.94-10.96	10.96-11.75	11.77-18.33	7.20-10.65	10.65-11.52	11.52-12.40	12.41-17.77
**ABSI**	0.058-0.075	0.075-0.081	0.081-0.088	0.088-0.152	0.049-0.077	0.077-0.083	0.083-0.090	0.090-0.141
**Conicity index**	0.920-1.169	1.171-1.283	1.283-1.381	1.382-2.154	0.830-1.230	1.230-1.326	1.326-1.420	1.421-2.050
**BRI**	1.29-2.97	2.98-4.33	4.34-5.71	5.71-13.75	1.50-4.09	4.09-5.48	5.49-7.19	7.19-14.83

Quartiles were derived separately within men and women using empirical distributions. Values show the observed range within each quartile. BMI, body mass index; WC, waist circumference; WHtR, waist-to-height ratio; WWI, weight-adjusted waist index; ABSI, a body shape index; BRI, body roundness index.

Supplementary Table S1B. Pearson correlation coefficients among traditional anthropometric parameters and novel body-shape indices among adults with type 2 diabetes mellitus.

Index	BMI	WC	WHtR	WWI	ABSI	CI	BRI
**BMI**	1.000	0.557	0.630	0.041	-0.287	-0.042	0.627
**WC**	0.557	1.000	0.942	0.781	0.606	0.783	0.931
**WHtR**	0.630	0.942	1.000	0.795	0.533	0.725	0.994

**WWI**	0.041	0.781	0.795	1.000	0.924	0.977	0.787
**ABSI**	-0.287	0.606	0.533	0.924	1.000	0.967	0.523
**CI**	-0.042	0.783	0.725	0.977	0.967	1.000	0.714
**BRI**	0.627	0.931	0.994	0.787	0.523	0.714	1.000

Matrix shows Pearson correlation coefficients. All correlations were statistically significant at $p<0.001$ except BMI with WWI ($p=0.133$) and BMI with conicity index ($p=0.120$). Abbreviations: BMI, body mass index; WC, waist circumference; WHtR, waist-to-height ratio; WWI, weight-adjusted waist index; ABSI, a body shape index; CI, conicity index; BRI, body roundness index.

Supplementary Table S2. Adjusted associations between sex-specific quartiles of traditional anthropometric parameters, novel body-shape indices and hypertension among adults with type 2 diabetes mellitus.

Index	Q1	Q2	Q2 p	Q3	Q3 p	Q4	Q4 p	p for trend
**BMI**	1.00 (reference)	0.99 (0.72-1.37)	0.961	1.45 (1.03-2.03)	0.033	2.31 (1.60-3.33)	<0.001	<0.001
**Waist circumference**	1.00 (reference)	1.50 (1.09-2.07)	0.014	1.85 (1.33-2.59)	<0.001	4.20 (2.84-6.20)	<0.001	<0.001
**WHtR**	1.00 (reference)	1.79 (1.30-2.47)	<0.001	2.30 (1.64-3.23)	<0.001	4.32 (2.95-6.31)	<0.001	<0.001
**WWI**	1.00 (reference)	2.01 (1.45-2.81)	<0.001	1.78 (1.29-2.48)	<0.001	3.12 (2.18-4.48)	<0.001	<0.001
**ABSI**	1.00 (reference)	1.26 (0.91-1.76)	0.165	1.45 (1.04-2.03)	0.029	1.75 (1.24-2.47)	0.001	0.001
**Conicity index**	1.00 (reference)	1.44 (1.04-2.00)	0.029	1.75 (1.26-2.45)	<0.001	2.37 (1.66-3.37)	<0.001	<0.001
**BRI**	1.00 (reference)	1.79 (1.30-2.47)	<0.001	2.30 (1.64-3.23)	<0.001	4.32 (2.95-6.31)	<0.001	<0.001

Odds ratios were estimated using logistic regression. Quartiles were sex-specific, with Q1 as the reference category. Models were adjusted for sex, age category, marital status, education, night eating habit, and physical activity. Hypertension was defined as systolic BP ≥140 mmHg and/or diastolic BP ≥90 mmHg, or current antihypertensive medication use.

Supplementary Table S3. Sex interaction tests for associations of traditional anthropometric parameters and novel body-shape indices with hypertension among adults with type 2 diabetes mellitus.

Index	Interaction term	Interaction OR (95% CI)	p-value
**BMI**	ZBMI x Female	0.85 (0.63-1.13)	0.264
**Waist circumference**	ZWC x Female	1.04 (0.79-1.37)	0.791

**WHtR**	ZWHtR x Female	0.98 (0.73-1.32)	0.914
**WWI**	ZWWI x Female	1.04 (0.79-1.36)	0.789
**ABSI**	ZABSI x Female	1.07 (0.83-1.39)	0.597
**Conicity index**	ZCI x Female	1.07 (0.82-1.40)	0.595
**BRI**	ZBRI x Female	0.96 (0.71-1.31)	0.807

Interaction terms were tested in logistic regression models including the anthropometric parameters, female sex variable, interaction term, age category, marital status, education, night eating habit, and physical activity. Hypertension was defined as systolic BP ≥140 mmHg and/or diastolic BP ≥90 mmHg, or current antihypertensive medication use. OR, odds ratio; CI, confidence interval.

Supplementary Table S4. Incremental predictive value of traditional anthropometric parameters and novel body-shape indices for identifying hypertension among adults with type 2 diabetes mellitus.

Added index	Block chi-square	df	p-value	Final Nagelkerke R^2	Adjusted OR (95% CI)	OR p-value
**BMI**	35.028	1	<0.001	0.142	1.50 (1.31-1.72)	<0.001
**Waist circumference**	61.566	1	<0.001	0.167	1.70 (1.48-1.95)	<0.001
**WHtR**	69.252	1	<0.001	0.174	1.79 (1.55-2.07)	<0.001
**WWI**	33.055	1	<0.001	0.140	1.46 (1.28-1.67)	<0.001
**ABSI**	11.550	1	<0.001	0.120	1.24 (1.09-1.41)	<0.001
**Conicity index**	26.721	1	<0.001	0.134	1.40 (1.23-1.60)	<0.001
**BRI**	66.149	1	<0.001	0.171	1.80 (1.55-2.09)	<0.001

Each index was added separately to a base model including sex, age category, marital status, education, night eating habit, and physical activity. Block chi-square refers to improvement in model fit after adding the anthropometric measure. OR, odds ratio; CI, confidence interval.

Supplementary Table S5A. Sensitivity analysis using expanded clinical hypertension definition: adjusted logistic regression.

Index, per 1-SD increase	Adjusted OR (95% CI)	p-value
**BMI**	1.82 (1.54-2.15)	<0.001
**Waist circumference**	2.00 (1.70-2.34)	<0.001
**WHtR**	2.22 (1.87-2.64)	<0.001
**WWI**	1.62 (1.39-1.89)	<0.001
**ABSI**	1.28 (1.11-1.48)	<0.001
**Conicity index**	1.51 (1.30-1.75)	<0.001
**BRI**	2.33 (1.93-2.81)	<0.001

The expanded clinical definition classified participants as hypertensive if they met the standard BP-or-medication definition or had documented hypertension in the medical record. Logistic regression models were adjusted for sex, age category, marital status, education, night eating habit, and physical activity.

Supplementary Table S5B. Sensitivity ROC analysis using expanded clinical hypertension definition.

Anthropometric measure	Cut-off	Sensitivity (%)	Specificity (%)	Youden	AUC (95% CI)	p-value
**BMI**	25.78	62.0	67.3	0.293	0.681 (0.650-0.713)	<0.001
**Waist circumference**	97.00	48.5	82.3	0.308	0.713 (0.683-0.744)	<0.001
**WHtR**	0.588	56.0	77.4	0.333	0.730 (0.700-0.760)	<0.001
**WWI**	10.50	79.0	48.0	0.270	0.662 (0.628-0.696)	<0.001
**ABSI**	0.079	68.0	49.5	0.176	0.596 (0.560-0.631)	<0.001
**Conicity index**	1.22	77.6	47.4	0.250	0.644 (0.610-0.679)	<0.001
**BRI**	5.16	56.0	77.4	0.333	0.730 (0.700-0.760)	<0.001

ROC analyses evaluated discrimination using the expanded clinical hypertension definition that additionally included documented hypertension in the medical record. AUC, area under the curve; CI, confidence interval.

Supplementary Table S6. Pairwise DeLong comparisons of AUC values between key anthropometric measures.

Group	Comparison	AUC 1	AUC 2	AUC difference	z	p-value	FDR-adjusted p-value
**Overall**	WHtR vs BMI	0.688	0.643	0.045	3.320	<0.001	0.001
	WC vs BMI	0.678	0.643	0.035	2.353	0.019	0.024
	BRI vs Conicity index	0.688	0.623	0.065	5.338	<0.001	<0.001
	BRI vs ABSI	0.688	0.581	0.106	6.742	<0.001	<0.001
	BRI vs WC	0.688	0.678	0.010	1.732	0.083	0.097
	BRI vs BMI	0.688	0.643	0.045	3.320	<0.001	0.001
	BRI vs WWI	0.688	0.635	0.053	4.993	<0.001	<0.001
	WHtR vs Conicity index	0.688	0.623	0.065	5.338	<0.001	<0.001
	WHtR vs ABSI	0.688	0.581	0.106	6.742	<0.001	<0.001
	WHtR vs WWI	0.688	0.635	0.053	4.993	<0.001	<0.001
	WHtR vs WC	0.688	0.678	0.010	1.733	0.083	0.097
	WHtR vs BRI	0.688	0.688	0.000	0.453	0.651	0.683
**Men**	BRI vs WC	0.692	0.674	0.018	2.078	0.038	0.057
	WC vs BMI	0.674	0.657	0.017	0.694	0.487	0.539
	BRI vs Conicity index	0.692	0.617	0.075	4.134	<0.001	<0.001
	BRI vs ABSI	0.692	0.576	0.116	4.929	<0.001	<0.001
	BRI vs WWI	0.692	0.640	0.053	3.338	<0.001	0.002
	BRI vs BMI	0.692	0.657	0.035	1.610	0.107	0.133
	WHtR vs BRI	0.692	0.692	0.000	0.000	1.000	1.000
	WHtR vs Conicity index	0.692	0.617	0.075	4.134	<0.001	<0.001
	WHtR vs ABSI	0.692	0.576	0.116	4.929	<0.001	<0.001
	WHtR vs WWI	0.692	0.640	0.053	3.338	<0.001	0.002
	WHtR vs WC	0.692	0.674	0.018	2.078	0.038	0.057
	WHtR vs BMI	0.692	0.657	0.035	1.610	0.107	0.133

**Women**	BRI vs ABSI	0.670	0.576	0.094	4.364	<0.001	<0.001
	BRI vs WWI	0.670	0.618	0.052	3.543	<0.001	0.001
	BRI vs WC	0.670	0.667	0.002	0.322	0.748	0.872
	BRI vs BMI	0.670	0.612	0.058	3.081	0.002	0.003
	WHtR vs BRI	0.670	0.670	0.000	0.154	0.878	0.921
	WHtR vs ABSI	0.670	0.576	0.094	4.364	<0.001	<0.001
	BRI vs Conicity index	0.670	0.614	0.055	3.350	<0.001	0.002
	WHtR vs WWI	0.670	0.618	0.052	3.543	<0.001	0.001
	WHtR vs WC	0.670	0.667	0.002	0.322	0.747	0.872
	WHtR vs BMI	0.670	0.612	0.058	3.081	0.002	0.003
	WHtR vs Conicity index	0.670	0.614	0.055	3.350	<0.001	0.002
	WC vs BMI	0.667	0.612	0.056	2.693	0.007	0.011

AUC, area under the receiver operating characteristic curve; FDR, false discovery rate. Pairwise AUC comparisons were conducted using DeLong tests for correlated ROC curves. FDR-adjusted p-values were calculated using the Benjamini-Hochberg procedure within each analysis group. The table presents selected comparisons involving WHtR, BRI, and WC because these measures showed the highest discriminatory performance in the main ROC analysis. WHtR and BRI comparisons should be interpreted cautiously because BRI is a monotonic transformation of waist circumference and height and was nearly identical to WHtR in ROC performance.

Supplementary Table S7. Comparison of optimal cut-off points derived using maximum Youden index and closest-to-(0,1) ROC criterion.

Group	Parameter	AUC	Youden cutoff	Youden sensitivity (%)	Youden specificity (%)	Youden index	Closest-to-(0,1) cutoff	Closest sensitivity (%)	Closest specificity (%)	Distance to (0,1)	Closest-method Youden index	Absolute cutoff difference
Overall	BMI	0.643	27.16	52.2	71.4	0.236	25.53	63.9	58.6	0.549	0.225	1.63
	WC	0.678	96.75	49.2	76.5	0.258	91.7	60.6	64.7	0.529	0.252	5.05
	WHtR	0.688	0.601	50.8	76.5	0.273	0.582	58.6	67.9	0.524	0.265	0.019
	WWI	0.635	10.5	79.7	43.0	0.227	10.87	70.8	50.2	0.577	0.211	0.37
	ABSI	0.581	0.083	49.1	64.2	0.133	0.083	50.4	62.6	0.621	0.13	0.0
	CI	0.623	1.239	74.6	45.1	0.197	1.284	61.7	55.3	0.589	0.17	0.045
	BRI	0.688	5.459	50.8	76.5	0.273	5.032	58.6	67.9	0.524	0.265	0.427
Men	BMI	0.657	27.31	43.8	84.7	0.285	25.37	56.6	71.1	0.522	0.276	1.94
	WC	0.674	84.5	70.4	56.3	0.267	88.95	61.5	64.7	0.522	0.263	4.45
	WHtR	0.692	0.529	71.7	58.9	0.307	0.529	71.1	59.5	0.498	0.305	0.0
	WWI	0.64	10.5	71.4	55.3	0.266	10.5	71.4	55.3	0.531	0.266	0.0
	ABSI	0.576	0.079	64.5	52.1	0.166	0.079	64.5	52.1	0.596	0.166	0.0
	CI	0.617	1.252	65.1	57.9	0.23	1.252	65.1	57.9	0.547	0.23	0.0
	BRI	0.692	3.917	71.7	58.9	0.307	3.923	71.1	59.5	0.498	0.305	0.007
Women	BMI	0.612	28.45	48.4	69.6	0.18	27.16	56.3	61.7	0.582	0.179	1.29
	WC	0.667	96.5	53.1	71.7	0.247	94.75	58.7	64.6	0.544	0.233	1.75
	WHtR	0.67	0.605	56.4	70.0	0.264	0.601	57.6	68.8	0.527	0.263	0.003
	WWI	0.618	11.87	42.6	75.4	0.18	11.36	59.5	54.6	0.609	0.141	0.51
	ABSI	0.576	0.083	51.3	60.4	0.117	0.083	51.3	60.4	0.628	0.117	0.0
	CI	0.614	1.366	44.7	72.9	0.176	1.334	52.6	64.6	0.592	0.172	0.032
	BRI	0.67	5.538	56.4	70.0	0.264	5.459	57.6	68.8	0.527	0.263	0.08

Abbreviations: AUC = area under the receiver operating characteristic curve; BMI = body mass index; WC = waist circumference; WHtR = waist-to-height ratio; WWI = weight-adjusted waist index; ABSI = a body shape index; CI = conicity index; BRI = body roundness index. Optimal cut-off points were estimated using two ROC-based criteria: the maximum Youden index, defined as

sensitivity + specificity − 1, and the closest-to-(0,1) criterion, defined as the cut-off with the shortest Euclidean distance from the ideal ROC point of 100% sensitivity and 100% specificity. Sensitivity and specificity are presented as percentages. The closest-method Youden index refers to the Youden index calculated at the cut-off selected by the closest-to-(0,1) criterion. The absolute cut-off difference was calculated as the absolute difference between the Youden-derived and closest-to-(0,1)-derived cut-off values, expressed in the original measurement scale of each anthropometric measure.

Supplementary Table S8. Sensitivity analysis of associations between anthropometric measures and hypertension using a common covariate adjustment set.

**Anthropometric Measure**	**Primary model, overall**	**Common-adjustment model, overall**	**Primary model, men**	**Common-adjustment model, men**	**Primary model, women**	**Common-adjustment model, women**
BMI	1.50 (1.31-1.72)	1.50 (1.30-1.72)	1.67 (1.30-2.14)	1.66 (1.29-2.13)	1.42 (1.20-1.69)	1.41 (1.19-1.67)
WC	1.70 (1.48-1.95)	1.70 (1.48-1.95)	1.67 (1.33-2.11)	1.67 (1.32-2.10)	1.73 (1.45-2.05)	1.72 (1.45-2.05)
WHtR	1.79 (1.55-2.07)	1.79 (1.55-2.07)	1.84 (1.44-2.35)	1.84 (1.44-2.35)	1.78 (1.48-2.13)	1.78 (1.48-2.13)
WWI	1.46 (1.28-1.67)	1.46 (1.28-1.67)	1.43 (1.15-1.78)	1.44 (1.16-1.80)	1.48 (1.25-1.75)	1.49 (1.25-1.77)
ABSI	1.24 (1.09-1.41)	1.24 (1.09-1.41)	1.19 (0.97-1.46)	1.20 (0.98-1.47)	1.27 (1.08-1.49)	1.28 (1.08-1.50)
Conicity index	1.40 (1.23-1.60)	1.40 (1.23-1.60)	1.34 (1.09-1.66)	1.35 (1.09-1.67)	1.44 (1.21-1.70)	1.44 (1.22-1.71)
BRI	1.80 (1.55-2.09)	1.80 (1.55-2.09)	1.87 (1.43-2.43)	1.87 (1.43-2.44)	1.77 (1.47-2.13)	1.77 (1.47-2.14)

Values are adjusted odds ratios (aORs) with 95% confidence intervals (CIs) per 1-SD increase in each anthropometric measure. Primary models used the prespecified stratum-specific covariate sets reported in the main analysis. For sensitivity analysis, a common

adjustment set comprising age category, marital status, education, income, glycemic control, night eating, and physical activity was applied to both sex-specific models; the overall model additionally included sex. Each anthropometric measure was entered separately. The common-adjustment analysis was conducted to assess the robustness of the findings to differences in covariate specification across sex strata. SD, standard deviation; BMI, body mass index; WC, waist circumference; WHtR, waist-to-height ratio; WWI, weight-adjusted waist index; ABSI, a body shape index; BRI, body roundness index.

Supplementary Table S9. Interaction between physical activity status and anthropometric measures in their associations with hypertension.

Measure	aOR, (95% CI), met WHO recommendation (n=1,166)	aOR, (95% CI), did not meet (n=190)	Ratio of ORs	P value (Wald)	P value (likelihood ratio)
BMI	1.57 (1.35-1.83)	1.09 (0.74-1.61)	0.72 (0.48-1.07)	0.100	0.107
WC	1.79 (1.54-2.08)	1.32 (0.90-1.93)	0.70 (0.48-1.02)	0.065	0.069
WHtR	1.87 (1.60-2.18)	1.49 (1.00-2.22)	0.75 (0.51-1.11)	0.153	0.160
WWI	1.48 (1.28-1.71)	1.49 (1.02-2.18)	0.91 (0.62-1.33)	0.623	0.624
ABSI	1.24 (1.08-1.42)	1.33 (0.92-1.93)	0.97 (0.67-1.41)	0.889	0.889
Conicity index	1.42 (1.23-1.64)	1.37 (0.95-1.98)	0.88 (0.61-1.27)	0.488	0.490
BRI	1.88 (1.59-2.22)	1.50 (1.00-2.24)	0.75 (0.50-1.11)	0.153	0.162

Adjusted odds ratios (aORs) and 95% confidence intervals (CIs) represent the association with hypertension per 1-SD increase in each anthropometric measure according to physical activity status. Physical activity was categorized according to whether participants met the WHO physical activity recommendation. Each interaction model included the standardized anthropometric measure, physical activity status, their product term, sex, age category, marital status, education, and night eating. The ratio of ORs represents the interaction effect comparing the anthropometric association among participants who did not meet the WHO recommendation with that among participants who met the recommendation. Interaction was assessed using Wald and likelihood-ratio

tests. WHO, World Health Organization; SD, standard deviation; BMI, body mass index; WC, waist circumference; WHtR, waist-to-height ratio; WWI, weight-adjusted waist index; ABSI, a body shape index; BRI, body roundness index.

Supplementary Table S10. Sensitivity analysis of anthropometric associations and discriminatory performance using the 2017 ACC/AHA hypertension threshold.

Anthropometric Measure	aOR (95% CI), primary definition	aOR (95% CI), ACC/AHA definition	AUC, primary	AUC, ACC/AHA
BMI	1.50 (1.31-1.72)	1.52 (1.27-1.81)	0.643	0.648
WC	1.70 (1.48-1.95)	1.71 (1.44-2.04)	0.678	0.688
WHtR	1.79 (1.55-2.07)	1.81 (1.51-2.17)	0.688	0.694
WWI	1.46 (1.28-1.67)	1.46 (1.24-1.72)	0.635	0.633
ABSI	1.24 (1.09-1.41)	1.25 (1.06-1.46)	0.581	0.576
Conicity index	1.40 (1.23-1.60)	1.40 (1.19-1.65)	0.623	0.622
BRI	1.80 (1.55-2.09)	1.89 (1.55-2.30)	0.688	0.694

For the sensitivity analysis, hypertension was defined according to the 2017 American College of Cardiology/American Heart Association (ACC/AHA) threshold as systolic blood pressure ≥130 mmHg and/or diastolic blood pressure ≥80 mmHg, or current antihypertensive medication use. The primary definition was systolic blood pressure ≥140 mmHg and/or diastolic blood pressure ≥90 mmHg, or current antihypertensive medication use. Adjusted odds ratios (aORs) and 95% confidence intervals (CIs) represent associations per 1-SD increase in each anthropometric measure. AUC values represent the discriminatory ability of individual anthropometric measures for identifying hypertension. Optimal cut-off points in Panel B were determined by maximizing Youden's index (sensitivity + specificity − 1) under the ACC/AHA hypertension definition. ACC/AHA, American College of Cardiology/American Heart Association; AUC, area under the receiver operating characteristic curve; SD, standard deviation; BMI, body mass index; WC, waist circumference; WHtR, waist-to-height ratio; WWI, weight-adjusted waist index; ABSI, a body shape index; BRI, body roundness index.
